# Delayed colonisation of *Acacia* by thrips and the timing of host-conservatism and behavioural specialisation

**DOI:** 10.1186/1471-2148-13-188

**Published:** 2013-09-09

**Authors:** Michael J McLeish, Joseph T Miller, Laurence A Mound

**Affiliations:** 1 Plant Geography Laboratory, Xishuangbanna Tropical Botanical Gardens, Chinese Academy and Sciences, Menglun, Mengla, Yunnan Province 666303, China; 2 Centre for Australian National Biodiversity Research, CSIRO Plant Industry, GPO Box 1600, Canberra, ACT 2601, Australia; 3 CSIRO Ecosystems Sciences, GPO Box 1700, Canberra, ACT 2601, Australia

## Abstract

**Background:**

Repeated colonisation of novel host-plants is believed to be an essential component of the evolutionary success of phytophagous insects. The relative timing between the origin of an insect lineage and the plant clade they eat or reproduce on is important for understanding how host-range expansion can lead to resource specialisation and speciation. Path and stepping-stone sampling are used in a Bayesian approach to test divergence timing between the origin of *Acacia* and colonisation by thrips. The evolution of host-plant conservatism and ecological specialisation is discussed.

**Results:**

Results indicated very strong support for a model describing the origin of the common ancestor of *Acacia* thrips subsequent to that of *Acacia*. A current estimate puts the origin of *Acacia* at approximately 6 million years before the common ancestor of *Acacia* thrips, and 15 million years before the origin of a gall-inducing clade. The evolution of host conservatism and resource specialisation resulted in a phylogenetically under-dispersed pattern of host-use by several thrips lineages.

**Conclusions:**

Thrips colonised a diversity of *Acacia* species over a protracted period as Australia experienced aridification. Host conservatism evolved on phenotypically and environmentally suitable host lineages. Ecological specialisation resulted from habitat selection and selection on thrips behavior that promoted primary and secondary host associations. These findings suggest that delayed and repeated colonisation is characterised by cycles of oligo- or poly-phagy. This results in a cumulation of lineages that each evolve host conservatism on different and potentially transient host-related traits, and facilitates both ecological and resource specialisation.

## Background

Host-plant specialisation is common and central to explanations for the enormous diversity of plant-feeding insects. Phytophagous insects vary in the taxonomic breadth of their respective host-plant range, but most still tend to use only a fraction of the plants available to them in their environment [1-5]. Generally, selection promoting both the broadening and reduction of host-plant resources must take place. Host-plant conservatism is not universal and selection for generalised host associations is expected to be persistent because of characteristics such as resource abundance variability or environmental predictability [6,7]. Colonisation of a new plant taxon signifies the broadening of a species host range, and specialisation on traits of the host show a narrowing of resource use. Explaining mechanisms that cause expansions or contractions in host-ranges has been difficult especially for species rich interactions [8] because the vagaries of time tend to obscure complex patterns of association [9]. Here we investigate the timing of colonisation by a lineage that evolved diverse specialised modes of resource-use but remained relatively species depauperate.

The enormous diversity of phytophagous insects has been attributed to traits associated with the insect herbivore (diet tolerances for plants and oviposition preferences), the plants they parasitise (defense strategies against herbivores), the interaction itself (‘coevolution’), ecological community interactions (predation & competition), or the environment (bottom-up forces). Conventional hypotheses posit ‘reciprocal’ or ‘sequential’ bitrophic interactions between traits of diversifying clades that drive insect and plant radiations [10-12]. Trade-offs in reproduction or diet, competition and predation, and tolerance to plant ‘defensive’ traits are central to these arguments [2,13]. Alternative explanations argue host-plant conservatism can be driven by predictability [14], climate [15], life-history characteristics [16], geographical contexts [17], plasticity [18], genetic predispositions or ecological compatibilities suited to the use of a resource [19], and host-range ecology [20] or genetics [21]. To distinguish among these causal mechanisms it is necessary to study evolutionary periods that are meaningful to the association of interest. Transitions to specialisation on a novel host-plant resource are only meaningful for a finite period because a shift to a narrower set of resources can be transient or bidirectional [3,8,22]. Determining the period that separates the origin of the insect group and the host-plants they feed on is essential to unraveling hypotheses explaining the origin or loss of narrow host ranges.

Discerning between colonisation and becoming reproductively isolated on the novel resource requires understanding distinct processes. The first phase in the evolution of a conservative host-plant affiliation is colonisation. Colonisation signifies a potential prelude to adaptation to a novel resource [5]. Colonisation of a novel plant lineage is either a fundamental shift to a resource previously not utilised in the evolutionary past or a secondarily derived association with a lineage used in the past [8]. The phylogenetic distance and dispersion among terminal host taxa has been used to distinguish between these two possibilities [23,24]. Furthermore, the relative time between the most recent common ancestor (MRCA) of the host lineage and inferred colonisation is expected to be indicative of the extent of the distance in resource space between natal and novel host [25]. This measure is informative because it describes the extent of niche-expansion, differences between alternative niches, and provides a framework for identifying trade-offs between them. The most direct means of testing this distance is to determine whether the common ancestors of insect and plant clades are contemporaneous or not. The second phase following initial contact leads to reproductive isolation on the new host that is assumed to ensue via disruptive selection in sympatry, or by gene flow disruption and drift in allopatric or parapatric isolation.

*Acacia* (*sensu stricto*) Mill. (Leguminosae, Mimosoideae) is broadly distributed over Australia with an estimated 1020 species. A fossil-calibrated molecular study has placed the origin of the legume subfamily Mimosoideae at approximately 42 Mya [26]. The fossil record indicates that species of subfamily Mimisoideae assignable to genera other than *Acacia* (*sensu lato*) [27] were present in the eastern Great Australian Bight approximately 37 Mya during the Oligocene. Australian *Acacia* is thus an immigrant taxon among a number of mimosoid genera and probably established in Australia during the Late Oligocene–Early Miocene [27]. The evidence suggests *Acacia* became a dominant part of sclerophyll communities in Australia during the Pliocene 7.0 – 1.5 Mya.

Thrips (Thysanoptera, Tubulifera, Phlaeothripinae) that parasitise Australian *Acacia* are uncharacteristic of the other 5500 estimated Thysanopteran species that mostly exhibit generalist relationships with plants [28]. Most of the Tubulifera species (*ca.* 60%, [29]) are fungivorous, some phytophagous, and fewer still are predators. Approximately 15% of the 2000 thrips species belonging to the Tubulifera are able to induce galls. Endemic northern tropical Australian thrips include species belonging to genera present in Southeast Asian in the wet tropics [30] suggesting thrips in Australia had an ancestral origin in a tropical environment. Thrips specialising on *Acacia* comprise several distinct behavioural suites that exhibit variation in host-specificity and oviposition strategies [31]. *Acacia* thrips, estimated to be in excess of 230 species [32], feed almost exclusively on sections *Phyllodineae* Pedley, *Plurinerves* Benth., and *Juliflorae* Benth. (*ca.* 397 spp., 216 spp., 255 spp. respectively, [33]). Of the 1020 *Acacia* species, approximately 950 develop phyllodes, the expanded petiole believed to be necessary for the radiation of thrips on *Acacia*. The most current molecular systematics of Thysanoptera supports the monophyly of this group [34]. The domicile-building *Acacia* thrips tie or glue phyllodes with silk to create a chamber. Kleptoparasitic thrips species invade and kill gall-inducing or domicile-building thrips on *Acacia* while opportunistic *Acacia* thrips species utilise the abandoned domiciles, galls, or similar constructions of other insect orders.

**Figure 1:**
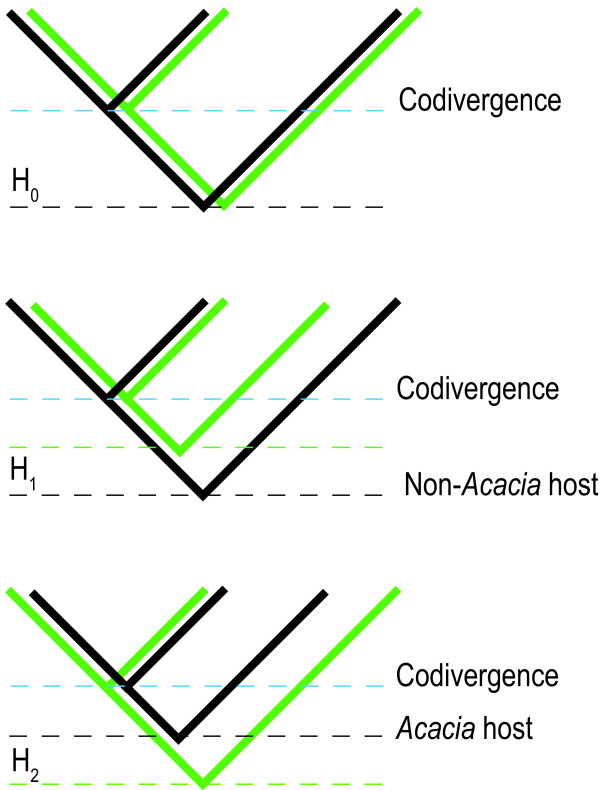
**Colonisation hypotheses.** Relative timing of the common ancestors of *Acacia* thrips in relation to *Acacia*. The green branches indicate the *Acacia* clade and the black branches thrips. We calibrated the relative timing of the two clades at the nodes where a parallel divergence (codivergence) event occurred between them [89]. Explanations for hypotheses: H_0_) contemporaneous origin of *Acacia* and *Acacia-*thrips MRCA; H_1_) multiple independent colonisations of *Acacia* and extinction of ancestral hosts; and H_2_) host shifting from more distantly related natal host.

Here we construct the most comprehensive *Acacia* (*sensu stricto*) molecular phylogeny to date and compare it with the evolutionary history of *Acacia* thrips. We expect one of three possible scenarios (Figure 1) explain the colonisation of *Acacia* by thrips, each with a distinct timing pattern. Phylogenetically contemporaneous common ancestors of insect and host plant are explained by insect lineages tracking the host with conserved host switching among related taxa. A pattern showing a considerably younger insect common ancestor compared to that of the host lineage is expected when a lineage that has not been used in its recent evolutionary past is colonised. An insect common ancestor that predates the host lineage requires invoking extinctions of insect lineages on other plant taxa or extinctions of distantly related ancestral host taxa. Specifically, we test the hypotheses that: i) the MRCA of *Acacia* thrips was contemporaneous with the MRCA of *Acacia* (rapid colonisation); ii) the MRCA of *Acacia* thrips postdates the origin of the MRCA of *Acacia* (delayed colonisation); or iii) predates the MRCA of *Acacia* (convergent colonisation and extinction). We interpret the results in terms of distinguishing between colonisation of *Acacia*, the evolution of host conservatism, and the evolution of ecological specialisation amongst thrips lineages with a focus on galling behaviour.

## Results

### Phylogenetic inference of *Acacia*

**Figure 2:**
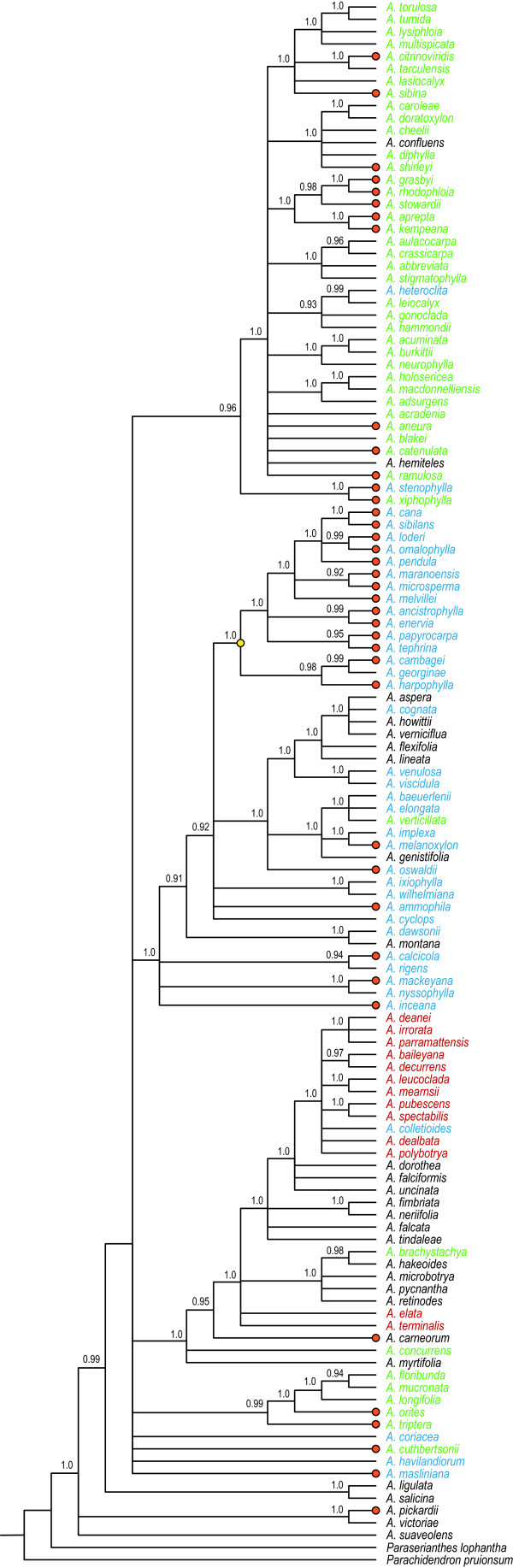
**Bayesian consensus tree of *Acacia.*** The consensus was derived from sampling every 1000^th^ tree of 100 × 10^6^ iterations with 2 chains and a GTR+I+Γ model applied to each gene locus and a burnin using 75,000 trees of a 100,000 tree posterior sample. Posterior probabilities > 0.90 are shown above branches. Red dots at branch terminals indicate host species. Taxon colour refers to *Acacia* sections: *Plurinerves* (blue); *Juliflorae* (green); *Phyllodineae* (black); and *Botrycephalae* (red).

We inferred phylogenies using parsimony-based and probabilistic approaches to evaluate uncertainty in topology, test deviations from taxonomic classifications, and generate a distribution of phylograms to be used in divergence time estimation (see below). The reliability of the inferences between independent Bayesian analyses was evaluated using the standard deviation of split frequencies that was below 0.01 on all runs. The potential scale reduction factor (PSRF) ranged from 1.000 to 1.012 for all parameters in the separate 100 × 10^6^ generations indicating consistent posterior parameters among runs. The Bayesian consensus tree indicated several poorly supported deeper nodes, but otherwise resolved the section clades (Figure 2). Our consensus tree showed good general agreement with section classifications [33]. The topologies of the parsimony, maximum likelihood, and Bayesian inferences all indicated very similar polyphyletic groupings of species from all four sections (Additional file 1, Additional file 2, Additional file 3, Additional file 4, Additional file 5 and Additional file 6). The SH-test for section monophyly indicated that 100 ML constraint trees generated using maximum liklehood were all significantly worse (*P* < 0.0001) than the topology of the Bayesian consensus phylogeny.

Considered together, species of sections *Phyllodineae* and *Juliflorae* cluster within the *Plurinerves* as do *Plurinerves* within the *Juliflorae*. *Acacia colletioides* (*Plurinerves*) groups within a clade that is otherwise comprised of section *Botrycephalae*. Section *Botrycephalae* is a derived clade of section *Phyllodineae*. *Acacia elata* and *A. terminalis* are paraphyletic with other *Botrycephalae* in section *Phyllodineae*. These topological associations are well supported in our Bayesian inference (Figure 2) and all other inferences (Additional file 4 and Additional file 5). *Acacia brachystachya* (*Juliflorae*) is well supported within section *Phyllodineae* in all inferences. Within *Juliflorae*, *A. stenophylla* (*Plurinerves*) is a well-supported sister-species of *A. xiphophylla* in all inferences. *Acacia heteroclita* and *A. confluens* (*Plurinerves*) also consistently grouped within the *Juliflorae*. Our inferences also show that section *Plurinerves* comprises *A. verniciflua*, *A. howittii*, *A. aspera*, *A. flexifolia*, *A. lineata*, *A. genistifolia*, and *A. montana*, which have all been classified as *Phyllodineae* species. These relationships were well-supported in the probabilistic inferences. *Acacia verticilata* (*Juliflorae*) grouped within the *Plurinerves* clade. Lineages that were not well resolved included *A. cuthbersonii*, *A. coriacea*, *A. masliniana* and *A. havilandiorum*, and the clade comprising *A. floribunda*, *A. mucronata*, *A. longifolia*, *A. orites*, and *A. triptera*.

### Phylogenetic inference of *Acacia* thrips

Inferences of *Acacia* thrips phylogeny were undertaken using the same procedure as for *Acacia*. The standard deviation of split frequencies was below 0.01 on all runs. The potential scale reduction factor (PSRF) was 1.000 for all parameters in the 100 × 10^6^ generations runs. The Bayesian consensus tree was largely concordant with that of previous work [32] and with our parsimony and likelihood inferences (Additional file 1, Additional file 2 and Additional file 3). An important difference in our topology arises due to the uncertain placement of *Kladothrips antennatus* in respect to the clade containing *Kladothrips zygus*. Previous phylogenetic inference [35] also shows poor support for this relationship despite more thorough testing of topology.

### *Acacia* divergence timing models

We inferred divergence time estimates using Bayesian and penalised likelihood (PL) approaches. The null molecular clock hypothesis of equal evolutionary rates was rejected (*P* < 0.0001). The estimated sample size (ESS) performance criteria (> 1000) indicated sufficient posterior parameter sampling. A total of *n* = 28 × 10^3^*Acacia* phylograms were filtered according to the topological constraint inferred with MrBayes. Of these, *n* = 25 *Acacia* PL chronograms were identical to the constraint. As this sample was not sufficient (age estimates not normally distributed) to calculate confidence intervals, we used the geometric mean to summarise the ranges of node age estimates inferred using PL. The dates of the parallel divergence inferred from the Bayesian approach and the geometric mean of the chronograms inferred using PL were 5.6 and 7.4 millions of years, respectively.

### *Acacia* thrips divergence timing models

We inferred timing estimates of *Acacia* thrips to generate and test relative divergence timing hypotheses (see below). The null molecular clock hypothesis was rejected (*P* < 0.0001). The ESS performance criteria (> 1000) indicated sufficient posterior parameter sampling. A total of *n* = 28 × 10^3^*Acacia* thrips phylograms were filtered according to a topological constraint inferred with MrBayes. Of these *n* = 10 were identical to the constraint. After scaling node ages, the dates for the MRCA of *Acacia* thrips were 14.38 mya under the Bayesian consensus, and 25.32 mya as the geometric mean calculated from the PL inferences.

### Testing between divergence timing models

**Figure 3:**
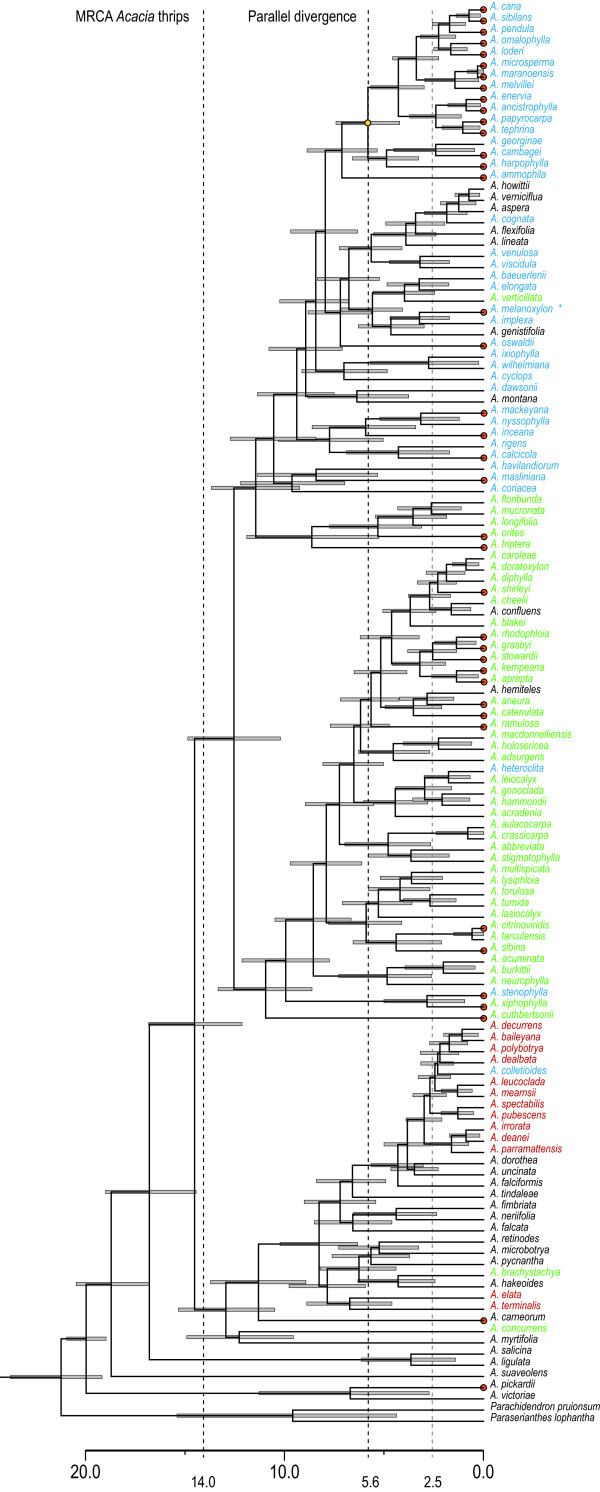
**Ultrametric comparison of *Acacia *thrips and *Acacia.*** An ultrametric Bayesian consensus tree of *Acacia* inferred from 75,000 post-burnin trees of the 100,000 trees in the Markov chain sample. A calibration date for the MRCA of *Acacia* of 20 Mya was the only prior used. Node bars indicate 95% highest posterior density intervals. Time scale in millions of years. Dashed lines indicate best-fit divergence timing model priors for thrips tree (14.0 & 5.6 MYA) and for a fossil of *A. melanoxylon during the Pliocene (2.5 – 5.0 MYA). Colour scheme as in Figure 2.

The range of divergence timing estimates represented in our BEAST and r8s inferences were summarised as divergence timing models (Table 1). The assumption of co-cladogenesis, contemporaneous MRCA’s at 20 Mya, and the maximal r8s estimate of approximately 50 million years for the MRCA were also tested. Bayes factor testing (Table 2) between divergence timing models using stepping-stone sampling of the log marginal likelihoods among our three hypotheses for the MRCA of *Acacia* thrips were: ln(H_14_) = −15934.63 and ln(H_20_) = −15934.94, (2*(ln(H_14_) – ln(H_20_)) = ln(BF_SS_) = 0.6); ln(H_25_) = −15936.31, (2*(ln(H_14_) – ln(H_25_)) = ln(BF_SS_) = 3.4); and ln(H_50_) = −15952.21, (2*(ln(H_14_) – ln(H_50_)) = ln(BF_SS_) = 35.2). The path sampling approach produced comparable results: ln(H_14_) = −15933.89 and ln(H_20_) = −15934.45, (2*(ln(H_14_) – ln(H_20_)) = ln(BF_PS_) = 1.16); ln(H_25_) = −15935.68, (2*(ln(H_14_) – ln(H_25_)) = ln(BF_PS_) = 3.6); and ln(H_50_) = −15952.00, (2*(ln(H_14_) – ln(H_50_)) = ln(BF_PS_) = 36.2). Both approaches indicate very strong support for the model where the MRCA of *Acacia* thrips occurred at approximately 14 Mya (Figure 3). The harmonic mean estimator of the marginal likelihood is only decisive when the ln(BF) is > 4.6. Bayes factors below this threshold should be interpreted with caution. However, the strength of the model decreases with divergence estimates older than 14 Mya under both approaches, indicating preference for the most recent estimate.

**Table 1:** Date priors for colonisation hypotheses

	**Acacia MRCA**	**Parallel split**	**Explanation**
H_14_	14.0	5.6	*Acacia* thrips MRCA after *Acacia* MRCA.
H_20_	20.0	5.6	*Acacia* thrips MRCA coincidental with *Acacia* MRCA.
H_25_	25.0	5.6	*Acacia* thrips MRCA before *Acacia* MRCA.
H_50_	50.0	5.6	*Acacia* thrips MRCA long before *Acacia* MRCA.

Date priors for the common ancestor of *Acacia* thrips and a parallel divergence event used to calibrate the thrips phylogeny. Path and stepping-stone sampling were used to estimate marginal likelihoods of each the divergence timing model for Bayes factor tests. A split in the thrips and *Acacia* phylogenies was treated as fixed [89].

**Table 2:** Bayes factor comparisons of timing models

**Hypothesis**	**Stepping-stone sampling**	**BF for H**_**14**_	**Path sampling**	**BF for H**_**14**_
ln(H_14_)	−15934.63	NA	−15933.89	NA
ln(H_20_)	−15934.94	0.6	−15934.45	1.2
ln(H_25_)	−15936.31	3.4	−15935.68	3.6
ln(H_50_)	−15952.21	35.2	−15952.00	36.2

Path and stepping-stone sampling was used to estimate the marginal likelihoods of divergence models inferred with BEAST. Bayes factor tests were made between the 14 Mya origin of *Acacia* thrips (best model = H_14_) and alternative timings based on penalised likelihood and Bayesian inferences. A BF indicates the strength of support for the best over the worst (ln) model.

## Discussion

Our findings indicate that the common ancestor of *Acacia* thrips postdates the common ancestor of *Acacia*. Putative absolute estimates of divergence timing indicate that thrips included *Acacia* in their host range approximately 14 Mya. We detected phylogenetic under-dispersion in host-species use that is consistent with i) cycles of oligophagy or polyphagy interspersed with repeated colonisations of *Acacia* over protracted periods before the evolution of resource specialisation; and ii) colonisation of host phenotypes that favour resource use in one environment over the other. Opportunistic and domicile-building thrips are polyphyletic groups whose common ancestors appeared between 5 and 10 Mya. The galling genus *Kladothrips* arose as recently as 6 Mya and represents the least uncertain shift to more stringent host-specificity by thrips and specialisation solely on *Acacia*. The common ancestors of the kleptoparasitic genus *Koptothrips*, and the gallers on whom they specialise, arose at approximately the same time*.* The putative date for the origin of the galling clade is of particular interest because several hypotheses posited for adopting this life history strategy can now be contextualised with the evolution of the Australian environment.

### Colonisation of *Acacia*

By definition, colonisation and the change to include a new species implies a broadening of host range and a period of oligophagy. Our ultrametric inference (Figure 3) for the transition between oligophagy (or polyphagy) at colonisation and host conservatism on *Acacia*, is explainable in several ways: i) oligophagy or polyphagy persisted for considerable evolutionary time after colonisation and before host conservatism on *Acacia* and the evolution of specialised behaviour; ii) host conservatism on *Acacia* evolved during or shortly after the colonisation of *Acacia* and specialised behaviour considerably later; or iii) host-conservatism at macro-evolutionary scales has obscured patterns of recolonisations of *Acacia* occurring at micro-evolutionary scales. Given the estimate for the origin of *Acacia* at 20 Mya, our results (Figure 3) indicate that the earliest possible transition to host conservatism on *Acacia* by thrips occurred at approximately 14 Mya. Uncertainty in our node estimates does not exclude earlier colonisation at 16.5 Mya.

Thrips colonised *Acacia* a considerable period after the host lineage radiated. Recolonisations might be expected to occur after initial contact with *Acacia* if there was an extended period before resource specialisation, and where host ranges include several plant taxa [20,36,37]. The ability to colonise a phylogenetically wider range of potential hosts is consistent with oligophagy and the relatively rapid colonisation of the *Juliflorae*, *Plurinerves*, and *Phyllodineae* (Figure 3). Ancestor lineages of these host sections existed before the MRCA of the gallers. This suggests host switching among distantly related species was initially accompanied by high species-specificity (e.g. [38]). There appears to be a protracted period before specialised behaviour evolved between thrips lineages and with *Acacia*. The 5 million year lag between the MRCA of the gallers and their divergence from the other genera is a relatively deep split. Primary and secondary associations with *Acacia* appear to be derived. Therefore, it is plausible that ancestors of extant species recolonised *Acacia* numerous times subsequent to the evolution of host conservatism on *Acacia*.

### Host conservatism in *Acacia* thrips

Conservative associations between an insect and host plant clade have been estimated at periods from 3 Mya (psyllids, [9]), 20 Mya (gallwasps, [39]), 40 Mya (yucca moths, [40]), to sometime since the Cretaceous (fig wasps, [16]). The two former studies of parasitic associations reported delayed colonisation of the host. The latter two associations infer co-cladogenesis with rapid colonisation scenarios and involve pollination mutualisms. Mutualisms are expected to select for more specific host conservatism due to the pollinator habit [41]. By comparison, parasitisms having relatively high species-specificity have been shown to involve switching between more distantly related plants [42]. Generally, host-range limits vary between antagonistic associations compared to symbioses and mutualisms where narrower species-to-species dependencies are more common [43-45]. Parasites of the galling habit exhibit wider host ranges than that of the prey species and evolve host-plant conservatism as a secondary association [46,47]. Kleptoparasitic and opportunist thrips have evolved associations with *Acacia* as secondary hosts presumably by targeting domiciles of other thrips species. Ecological specialisation amongst *Acacia* thrips characterized by primary and secondary associations predict forces selecting for host-plant conservatism will vary among the *Acacia* thrips as do their host-plant ranges.

Host conservatism is transitory as diet breadths of phytophagous insects fluctuate over time [8,24,48-50]. Thrips with strict host plant associations are rare, exhibit a willingness to engage in feeding on a wide variety of plant families, and have similar feeding apparatus in all life stages [51]. This suggests plasticity in host plant tolerance is possibly linked to secondary associations with food resources that have been used in the evolutionary past [48,52,53] and facilitated cycles of recolonisation. Gall-inducing thrips, apart from those on *Acacia*, are able to exploit multiple plant taxa [25]. This suggests host choice by thrips involves multiple evolutionarily labile traits. For example, galling by sawflies has arisen independently on multiple occasions across five plant families [54]. The nematine subfamily of sawflies that specialises on the genus *Salix* also has several origins of galling, but on various parts of the plant [55]. Therefore, trade-offs between plesiotypic trait compatibility among available plants [19] and selection for traits resulting in host conservatism, should strongly favour thrips associations with *Acacia*. In other words, a broad diet breadth facilitates colonisation of new plant lineages, but selection for host conservatism develops when genetic trade-offs in performance arise on the new host.

### Host conservatism and environment

Shifting to a new host plant can result from trade-offs between alternative environments associated with natural enemies [56,57] and larval or oviposition performance on alternative hosts [58-61]. Thrips colonised *Acacia* at a time when it presumably supported a similar diversity of insects as it does today [62]. As a result, thrips likely experienced fitness costs associated with predation or competition during colonisation. Furthermore, host conservatism and ecological specialisation expressed by contemporary thrips species appears to have taken several millions of years to evolve as Australia experienced pronounced environmental change and ecological disruption. Our estimates suggest that the common ancestors of the thrips behavioural suites arose approximately at the beginning of the Quaternary when Australia’s climate was strongly linked to glacial/interglacial cycling [63]. Before this period, Australia experienced a more general transition from humid to seasonal climates. The development of arid environments resulted in profound structural changes to animal and plant communities [64,65] including *Acacia*[66,67]. Performance between host lineages has been shown to respond to such habitat gradients [15,68]. Our timeline for the colonisation of *Acacia* coincides with the first major step towards aridity during the mid-Miocene and the development of a more acute dry season in Central Australia [63]. It is at this time that *Acacia* replaced *Eucalyptus* in the developing arid regions. *Acacia* represented an expanding resource with geographical range changes that potentially influenced host resource suitability and predictability.

Host conservatism evolved under transient abiotic and biotic conditions in a non-random manner during the diversification of *Acacia* thrips. Our inferences indicate that the phylogenetic distribution of *Acacia* host-species compared to non-hosts is non-random. For example, one or several species of *Acacia* in crown clades that support thrips have intermixed lineages that are absent of thrips parasitism. This form of phylogenetic under-dispersion, where host lineages are distantly related and intermixed among terminal branches, is characteristic of recolonisation episodes [23,24,48]. We suggest these patterns are robust to our incomplete sample. *Acacia* thrips are a species poor group (*ca*. 235 spp., [32]) compared to *Acacia* (> 1000 spp.). Single species of *Acacia* are known to support upto 5 thrips species, reducing the realised number of host species even further. Similarly, at a very broad taxonomic scale, thrips radiations on several plant families have occurred with noticeable absences from others. Thrips are associated with several angiosperm families including species of *Ficus* (Moraceae), *Geijera parviflora* (Rutaceae), and *Casuarina* (Casuarinaceae) as well as genera specific to mosses, conifers, and cycads [30,69,70]. Plant families with very few or no specific patterns of affiliation with thrips include Myrtaceae, Proteaceae, Asteraceae, Leguminosae, and Poaceae. The latter two families have a remarkable diversity of thrips species attracted to flowers and leaves respectively, but with no perceivable pattern of affiliation. We propose that these phylogenetic patterns of under-dispersion are indicative of host conservatism driven by biotic and abiotic environmental compatibilities subsequent to delayed colonisation.

### Host conservatism and geographic distribution

Thrips lineages associated with phylogenetically isolated host species suggests geographic range characteristics of non-host sister-taxa are not suited to supporting *Acacia* thrips [71]. *Acacia* have typical geographical range distributions with most species having small and intermediate range sizes and few with large distributions. The size of the host species geographic distribution appears independent of extant thrips associations, but might not be indicative of ancestral ranges during colonisation. For example, *A. oswaldii* is a broadly distributed arid-zone species inhabited by galling and kleptoparasitic thrips species. *Acacia oswaldii* is phylogenetically distinct from sister-taxa that are not parasitised by thrips (Additional file 6) suggesting this host has geographic range characteristics suited to the maintenance of *Acacia* thrips populations while sister-species do not. *Acacia cuthbersonii*, *A. carneorum*, and *A. pickardii* are also phylogenetically isolated, but these species have broad as well as very narrow geographic range distributions among them. Phylogenetically isolated clades supporting thrips that include *A. triptera*, *A. kempeana*, *A. aneura*, *A. citrinoviridis*, and *A. xiphophylla* also have species with broad and narrow geographic distributions, suggesting historical factors are important to maintenance of *Acacia* thrips populations. The *Acacia* lineage possessing both species with phyllodes (section *Phyllodineae*) as well as those with bipinnate leaves (section *Botrycephalae*) are all presumably unsuitable for thrips inhabitation due to geographic range characteristics, biotic associations, or heritable traits. Phyllodinous *Acacia* are phylogenetically and chemically related to bipinnate forms [72-74], providing some basis for the presence of heritable traits partly explaining thrips absence in this stem clade (but see below). We suggest these patterns are consistent with host conservatism among genetically similar and dissimilar hosts with heritable and non-heritable characteristics favouring host use.

### Ecological specialisation on *Acacia*

Host conservatism and host specialisation can be differentiated as the evolutionary conservative association of thrips and *Acacia*, and the evolution of distinct phenotypes that emerge directly or indirectly as a result of host conservatism [2]. *Acacia* thrips exhibit diverse phenotypes that characterise distinct forms of host specialisation that appear to have evolved in a cumulative manner on particular *Acacia*-related traits. Selection pressures and new ecological opportunities for specialisation arising during the course of climate transition should be dependent on stochastic and plesiotypic factors. Our timeline for the origins of *Acacia* thrips genera suggests the evolution of ecological specialisation was approximately contemporaneous, occurring midway between the common ancestor and the present. Once the inclusion of a novel host-plant in the dietary range of an insect occurs, conservative interactions conceivably select for traits such as gall induction [75]. Our findings show corresponding origins of domicile-building and kleptoparasitic thrips genera that are consistent with previous work [76]. It was suggested that gall-inducing was a selective response to kleptoparasitism. The observation that facultative kleptoparasitsm is present in some species of *Koptothrips* suggests an intermediate stage of specialisation similar to opportunism that has responded to selection on habitat. These observations make it difficult to determine whether behavioural specialisation by kleptoparasitic lineages on the domicile-building and gall-inducing thrips evolved as a consequence of either biotic or abiotic pressures.

Abiotic forces have a strong influence over trade-offs between heritable and non-heritable constraints on host use [15]. The physical environment and spatial context of hosts has been shown to structure insect communities [77,78]. For instance, the impetus for galling behaviour is believed to include non-mutually exclusive factors associated with avoidance of natural enemies [79], minimising environmental stress [80], or optimising nutritional choices [81]. Galling arose in *Kladothrips* near the Miocene-Quaternary boundary*.* Pronounced ecological transitions during the Quaternary would have changed the selective landscape in Australia. Evidence of non-random host associations such as phylogenetic under-dispersion is also indicative of specialised behaviour as a response to habitat and resource selection [77]. This type of host-plant conservatism suggests colonisation of phenotypes that favour host-use in one environment over the other (e.g. [82]). For instance, the evolution of galling is believed to be favoured in harsh xeric environments [83] that became particularly pronounced in Australia during the Quaternary. However, *Acacia* thrips are more species rich and occupy more diverse ecological roles outside the arid biome. At the other climatic extreme, *Acacia* thrips are absent from hydric habitats in southeastern Australia [31,84]. Social behaviour in *Kladothrips* arose with the evolution of a specialised defensive caste and is symptomatic of species distributed in non-arid areas. An alternative strategy exhibited by non-social *Kladothrips* species is the adoption of physogastry and extreme fecundity. This ‘boom-or-bust’ lifestyle tends to characterise arid-distributed species. These hypotheses remain untested. Behavioural differentiation between environments predicts that ecological specialisation under conditions alternating between xeric and mesic environments, was based on selection on behaviour and habitat specialisation.

## Conclusions

A considerably younger *Acacia* thrips common ancestor compared to that of *Acacia* is consistent with colonisation of a lineage that has not been used as a host in its recent evolutionary past. Presumably either oligophagous or polyphagous ancestral thrips populations were able to feed on and recognise *Acacia* subsequent to the evolution of host conservatism. We propose that colonisation of *Acacia* was initially characterised by either oligophagy or polyphagy and subsequent recolonisations by a number of ancestral lineages. Colonisation of phenotypically and environmentally suitable lineages occurred over a protracted period that resulted in phylogenetically under-dispersed pattern of host conservatism. The evolution of host conservatism on suitable *Acacia* lineages facilitated the evolution of ecological specialisation during a period that coincided with aridification and ecological disturbance in Australia. Our findings support the hypothesis that host conservatism is a process shaped by changing abiotic and biotic forces, and ecological specialisation an additive process imposed by changing selective pressures on habitat preference and behaviour.

## Methods

### Phylogenetic inference of *Acacia*

We inferred *Acacia* and thrips phylogenies using parsimony and probabilistic approaches to assess topological support for both thrips and *Acacia* datasets and to generate a distribution of phylograms to be used in divergence time estimation (see below). Four plastid loci (*matK*, *rpl32-trnL* intergenic spacer, *psbA-trnH* intergenic spacer, and *trnL-F* intron and intergenic spacer) and two nuclear loci from internal and external transcribed spacers (*ITS* and *ETS* respectively) of *Acacia* were sequenced. Previous work [85] has inferred several smaller trees that included multiple exemplars of some species used in this study. Primers and PCR protocols are described in a previous study [86]. We combined new sequence data with single representatives of species from the previous study and added 61 new species that included all *Acacia* that thrips are known to specialise on*.* Together our sample comprised 125 (12.6%) described species and two outgroup taxa. We used *Paraserianthes lophantha*[87], the sister taxon of *Acacia*, and *Parachidendron pruionsum*[85] as outgroup taxa.

Probabilistic and parsimony inferences were conducted in MrBayes v.3.2.1 [88], RAxML v.7.3.0 [89], and MEGA5 v.5.05 [90]. We used jModelTest v.2.1.1 [91] to justify priors for models of sequence evolution that were selected according to the Akaike and the Bayesian Information Criteria (AIC & BIC; [92]). The best-fit model selected by the AIC or BIC test for each of the plastid and nuclear plant often differed (Additional file 7: Table S1). Most of these models could not be specified in the divergence time estimation approaches, so we applied the general time reversible (GTR) model that generates distributions of parameters that approximate sub-models of the GTR model [93]. For the MrBayes and RAxML approaches, we fitted separate model priors (GTR with a proportion of invariant sites (I) and gamma (Γ) distributed rates) to each of the plastid and nuclear loci. Each Bayesian inference was performed over two simultaneous analyses with two Markov chains. Analyses were run four times to verify the repeatability of the phylogenetic inference; two runs at 100 × 10^6^ and two at 40 × 10^6^ generations. Posterior probabilities were derived from 75,000 trees sampled from post-burnin generations 25–100 million, after the chains had reached apparent stationarity. Convergence was assessed using the MCMC Tracer Analysis Tool v.1.5 [94] by plotting the log likelihoods to assess the point in the chain where stable values were reached. For the likelihood analyses conducted with RAxML, we implemented the rapid bootstrap analysis and search for the best-scoring tree using 1 × 10^4^ runs. Our parsimony analyses conducted with MEGA5 were implemented using the Close-Neighbor-Interchange (CNI) method with random starting tree and 1 × 10^3^ bootstrap replicates.

**Figure 4:**
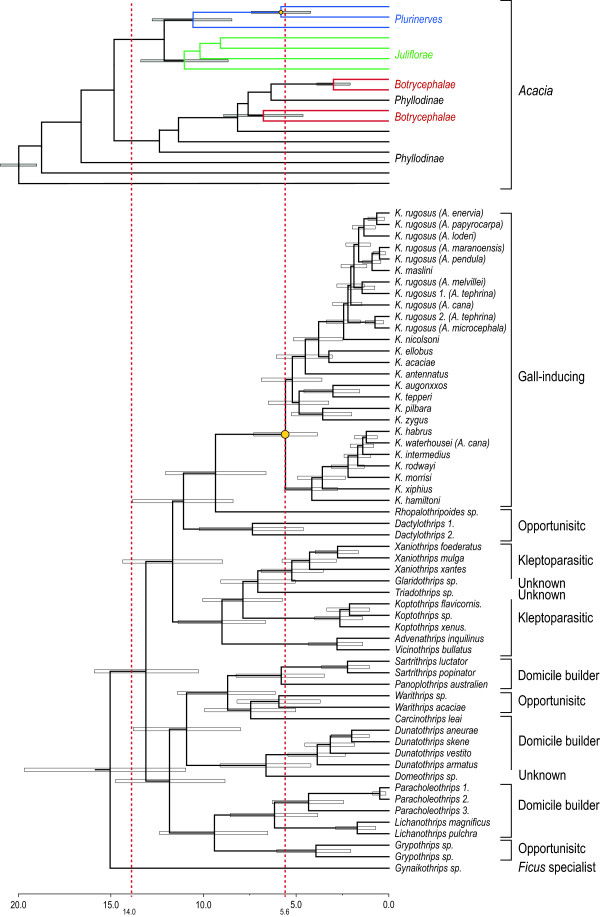
**Divergence timing of *Acacia.*** An ultrametric comparison between *Acacia* (above) and thrips (below). *Acacia* tree is abbreviated and colour branches indicate host sections *Plurinerves* (blue), *Juliflorae* (green), *Phyllodineae* (black), and *Botrycephalae* (grey). Time scale in millions of years is based on molecular dating [103]. Horizontal bars on nodes indicate the 95% highest posterior density intervals. Red dashed lines indicate the calibration model priors used for the thrips inference. Behavioural categories of thrips indicated on the right. The yellow dot indicates the node when the inferred parallel divergence occurred.

Current phylogenetic relationships of Australian *Acacia* are not consistent with past classifications [85,86]. In our data, *Acacia diphylla* is a synonym of *Acacia blakei* subsp. *diphylla* (Section *Juliflorae*). Although recent revisions have placed older classifications into doubt, we used the commonly used taxonomic ranking of Pedley. Four main clades including sections *Plurinerves*, *Juliflorae*, *Botrycephalae*, and *Phyllodineae*[33] were considered in this study. We used the SH-test [95] as implemented in RAxML, to assess the section classifications presented in Maslin (2004) against our consensus trees. We specified a constraint tree that grouped each section as a multifurcating clade using Mesquite v.2.75 [96]. The constraint tree consisted of three polytomous crown clades each grouping the section classifications *Juliflorae*, *Plurinerves*, and *Botrycephalae*, and a fourth stem clade as the *Phyllodineae*. We used RAxML to resolve the multifurcations and optimise the topology under maximum likelihood given the sequence alignment and gene partitions. The test used 100 runs (generates 100 ML trees) and the GTR+I+Γ substitution model. Each of the resulting 100 bifurcating topologies were compared with our consensus using the SH test (Figure 4).

### Phylogenetic inference of *Acacia* thrips

The sole dependence of extant species of *Acacia* thrips on *Acacia* might be taken as evidence for the common ancestor sharing this attribute. However, without fossil material this is difficult to test and might not be the case given the difficulty in accurately estimating ancestral host-ranges. Previous work [32] inferred an *Acacia* thrips phylogeny using most of the data presented here. The classification of the galling species has since been revised. Three genera comprising the galling species have been collapsed into the genus *Kladothrips*. We have added new samples of the galling species. Furthermore, the *Kladothrips rugosus* species complex previously believed to be an oligophagous group, are now considered separate monophagous species. Species delimitation using molecular approaches has been conducted in previous work [97] and demonstrates genetic divergence thresholds between these lineages are characteristic of separate species. As such, species of this clade are undescribed and have been designated by their host species association. Sequence data of *cytochrome oxidase one* (*COI*) mitochondrial DNA, and nuclear loci *elongation factor one alpha* (*EF-1α*) and *wingless* gene fragments [98] were used to reconstruct a thrips phylogeny. Full details describing primers, PCR conditions, sequencing, alignment, and substitution model priors can be found in [96]. The outgroup taxon *Gynaikothrips* specialises as a leaf-galler on the genus *Ficus*[70] and was chosen based on previous work [32].

The same phylogenetic and model testing approaches conducted with the *Acacia* sequence data was repeated using the thrips data. For the MrBayes and RAxML approaches, we fitted separate model GTR+I+Γ to the 1^st^, 2^nd^, and 3^rd^ codon positions of *COI* and single partitions of *EF-1α* and *wingless.* The thrips Bayesian inferences were performed using four Markov chains. The same protocols for assessing repeatability and stationarity of *Acacia* inferences were applied.

### *Acacia* divergence time estimation

We tested whether our *Acacia* consensus tree obeyed a molecular clock hypothesis using MEGA5 by comparing the ML value for our topologies with and without the molecular clock constraints using the GTR+I+Γ model of evolution. Ultrametric trees were inferred using PL as conducted in r8s v.1.8 [99] and a Bayesian approach conducted in BEAST v.1.7.2 [100]. Date calibrations were based on the most recent divergence timing estimates [101] with the MRCA of *Acacia* (*sensu stricto*) at between 14.6 and 21.2 Mya. We used a putative date of 20 million years before present as a fixed calibration for the origin of *Acacia*. This calibration prior was fixed to facilitate testing the relative timing between the two clades. Absolute divergence dates based on previous estimates are assumed to broadly contextualise *Acacia* divergence timing with changes in the Australian environment. A macrofossil of an extant species *Acacia melanoxylon* identified from the Pliocene [27] enabled us to compare our inferred dates with that of the fossil record.

Maximum clade credibility trees were inferred using BEAST. The model of evolution used to infer divergence time estimates was based on the priors implemented in the MrBayes inference across the locus partitions: GTR+I+Γ, four gamma categories, and empirical base frequencies. The chain was run for 100 × 106 generations and sampled every 1000^th^ generation and the last 75,000 trees used for inferring ultrametric consensus trees and 95% highest posterior density intervals. We conducted several pilot runs using different priors on gene partitioning, topology constraints, and parameter distributions to estimate clock rates to use as priors in the dedicated runs in order to meet the posterior ESS optimisation criteria. We used the lognormal relaxed clock (with ‘estimate rate’) for the gene partitions and a normal distribution prior for the ‘ucld.mean’ for all partitions. The Yule process was used as the speciation model with a starting ultrametric tree topology constraint from a pilot BEAST inference that used a PL tree generated with r8s. Substitution and clock models were set to unlinked across gene partitions, and linked for tree priors. We used date priors only for those topology constraints necessary to define an ingroup and to calibrate the tree for the ultrametric hypothesis comparisons tested using path and stepping-stone sampling methods (see below). The ‘stem’ function was activated and clades assumed to be monophyletic. The r8s PL approach uses a data-driven cross-validation procedure to select an appropriate level of rate smoothing given branch length estimates proportional to substitution differences.

### *Acacia* thrips divergence time estimation

Divergence time estimates for the *Acacia* thrips were inferred using a nominal root age of 1. As no reliable date calibration prior for the origin of the most recent common ancestor of *Acacia* thrips was available, we preferred to scale the thrips trees in respect to the date of a parallel divergence event [98] and our *Acacia* ultrametric trees generated by BEAST and r8s (see below). Although the parallel divergence was our only reliable date prior, the use of a single, derived calibration can produce spurious root-node age estimates. The Yule process was used as the speciation model with a starting ultrametric tree topology constraint from a pilot BEAST inference that used default priors. Otherwise, the same procedures used to estimate *Acacia* divergence timing were implemented with the thrips data.

### Tests of temporal & phylogenetic congruence

We used a bifurcation in both the *Acacia* and thrips clades that is an inferred point of parallel diversification, and therefore temporarily concordant, to estimate the relative timing between clades. To test whether the MRCA of *Acacia* thrips was contemporaneous with, pre-, or post-dated the MRCA of *Acacia* we compared ultrametric tree inferences using the various date priors estimated for their MRCA’s and the parallel divergence. All posterior trees from our thrips and *Acacia* MrBayes runs were filtered using a consensus topology constraint conducted in PAUP* v.4b10 [102], and divergence times estimated from these phylograms using r8s. Dates for the parallel bifurcations of respective social and non-social thrips clades parasitising the same sister host-clades [98] were used to scale the *Acacia* thrips chronograms to estimate the date of the MRCA. The parallel diversification of *Acacia* thrips on the stem clade comprising *A. cambagei* and *A. harpophylla* and species in the sister-clade was used as a date prior to match with the divergence date of these hosts in the *Acacia* chronograms. The divergence timing estimates generated from BEAST and r8s provided a maximum and minimum date priors for the origin of the MRCA of *Acacia* thrips in respect to the MRCA of *Acacia*. We tested these timing hypotheses as well as the assumption of co-cladogenesis (Table 2). The different divergence timing models were compared using Bayes factors (BF) by estimating marginal likelihoods using path sampling and stepping-stone sampling conducted in BEAST [103,104]. In terms of the relative strength of the model, the ln(BF) (natural log) indicates: strong (2.3-3.4), very strong (3.4-4.6), and decisive (> 4.6 ) evidence [105].

## Availability of supporting data

Complete sequences of the *Acacia* specimens have been submitted to Genbank (http://www.ncbi.nlm.nih.gov/genbank/) [accession numbers JF419907-JF420546]. Thrips sequences are available from GenBank [accession numbers AF448280-AF289019, AF386676-AF386737, AY827474-AY827481, AY920988-AY921000, AY921058-AY921069, and DQ246453-DQ246516].

## Competing interests

The authors declare that they have no competing interests.

## Authors’ contributions

The conception and design of the investigation was developed by MJM. All authors contributed to field collections. Bench work conducted by JTM & MJM. Sequence alignments conducted by JTM and MJM and data analyses were undertaken by MJM. Interpretation of results and manuscript drafting were contributed to equally. All authors have read and approved the final manuscript.

## Supplementary Material

Additional file 1 **Bayesian consensus tree of *Acacia *thrips.** A Bayesian consensus tree of *Acacia* thrips inferred using MrBayes inferred from the last 75,000 trees of the 1000,000 tree posterior distribution. Posterior probabilities > 0.90 are shown on branches. Grey dot indicates parallel divergence split. Click here for file

Additional file 2 **Parsimony inference of *Acacia *thrips.** A parsimony consensus tree of *Acacia* thrips inferred using the Close-Neighbor-Interchange (CNI) method with 1,000 bootstrap replicates. Node support is shown on branches. Grey dot indicates parallel divergence split. Click here for file

Additional file 3 **Likelihood bootstrap consensus of *Acacia *thrips.** A maximum likelihood consensus tree of *Acacia* thrips inferred with RAxML using the rapid bootstrap search algorithm with the CNI method and 10,000 bootstrap replicates. Node support is shown on branches. Grey dot indicates parallel divergence split. Click here for file

Additional file 4 **Parsimony bootstrap consensus of *Acacia.*** A parsimony consensus tree of *Acacia* inferred using the rapid bootstrap search algorithm with the CNI method and 1,000 bootstrap replicates. Node support is shown on branches. Yellow dot indicates parallel divergence split and red dots a host species. Taxon colour refers to *Acacia* sections: *Plurinerves* (blue); *Juliflorae* (green); *Phyllodineae* (black); and *Botrycephalae* (red). Click here for file

Additional file 5 **Maximum likelihood bootstrap consensus of *Acacia.*** A maximum likelihood consensus tree of *Acacia* inferred with RAxML using the rapid bootstrap search algorithm with the CNI method and 10,000 bootstrap replicates. Node support is shown on branches. Yellow dot indicates parallel divergence split and red dots a host species. Taxon colour refers to *Acacia* sections: *Plurinerves* (blue); *Juliflorae* (green); *Phyllodineae* (black); and *Botrycephalae* (red). Click here for file

Additional file 6 **Bayesian consensus phylogram of *Acacia.*** A Bayesian consensus phylogram of *Acacia* inferred from the last 75,000 trees of the 1000,000 tree posterior distribution. Branch lengths proportional to substitution differences. Yellow dot indicates parallel divergence split and red dots a host species. Taxon colour refers to *Acacia* sections: *Plurinerves* (blue); *Juliflorae* (green); *Phyllodineae* (black); and *Botrycephalae* (red). Click here for file

Additional file 7 **Substitution model estimates.** Best-fit models for the *Acacia* and *Acacia* thrips sequence data were estimated across gene and codon locus partitions using jModelTest according to the Akaike and the Bayesian Information Criteria (AIC & BIC). Gamma distributed rates = G; invariant proportion of sites = I. Click here for file
