# Supplementary material for: Delayed colonisation of Acacia by thrips and the timing of host-conservatism and behavioural specialisation
Source: BMC Evol Biol. 2013 Sep 9;13:188. doi: 10.1186/1471-2148-13-188 (PMC3846595; doi:10.1186/1471-2148-13-188)
Supplement: Additional file 7 — Substitution model estimates. Best-fit models for the Acacia and Acacia thrips sequence data were estimated across gene and codon locus partitions using jModelTest according to the Akaike and the Bayesian Information Criteria (AIC & BIC). Gamma distributed rates = G; invariant proportion of sites = I. [file 1471-2148-13-188-S7.pdf]

**Partition**	**AIC Best-fit**	**BIC Best-fit**
Thrips		
COI	TPM1uf+I+G	TPM1uf+I+G
COI 1st codon	TIM2+I+G	TrN+I+G
COI 2nd codon	TPM1uf+I+G	F81+I+G
COI 3rd codon	TPM1uf+G	TPM1uf+G
EF-1a	TIM2ef+I+G	TIM2ef+G
wingless	GTR+G	TrN+G
Acacia		
ETS	GTR+G	TIM3+G
ITS	TVM+I+G	TPM2uf+I+G
rpl32-trnL	TPM2uf+I+G	TPM2uf+G
matK	TIM2+G	HKY+G
psbA-trnH	TVM+G	TVM+G
trnL-F	TPM1uf+G	TPM1uf+G
